# Comparison of the predictive value of progesterone‐related indicators for pregnancy outcomes of women undergoing the short‐acting GnRH agonist long protocol: a retrospective study

**DOI:** 10.1186/s13048-021-00768-2

**Published:** 2021-01-12

**Authors:** Yangyang Zhang, Yang Xu, Yuqiong Wang, Qing Xue, Jing Shang, Xiuli Yang, Xuemin Shan

**Affiliations:** Department of Obstetrics & Gynecology, Peking University First Hospital, 100034 Beijing, China

**Keywords:** Progesterone, P/E_2_, P‐to‐follicle index, P‐to‐mature oocyte index, Pregnancy outcome

## Abstract

**Background:**

There are many progesterone (P) elevation-related indicators for predicting pregnancy outcomes, including the serum P, P-to-oestradiol ratio (P/E_2_), P-to-follicle index (PFI), and P-to-mature oocyte index (PMOI); however, due to inconsistencies in study populations and controlled ovarian hyperstimulation (COH) protocols among studies, these indicators are controversial. Moreover, no researchers have included these four commonly used indicators in one study to compare their predictive efficacies. The objective of this study was to compare the predictive value of P-related indicators for pregnancy outcomes of women undergoing the short-acting GnRH agonist long protocol.

**Methods:**

A total of 612 infertile women undergoing IVF/ICSI were recruited for this study. Serum samples were obtained on the morning of HCG injection for serum P and E_2_ measurements. Transvaginal ultrasound was performed to determine the follicle count (≥ 14 mm in diameter). The number of mature oocytes was observed in the embryo laboratory after oocyte retrieval.

**Results:**

In cases of *P* < 2.5 ng/ml, there was no significant difference in the serum P level or P/E_2_ between the pregnant group and the non-pregnant group. The PFI and PMOI of the pregnant group were significantly lower than those of the non-pregnant group. According to the stratified analysis of the ovarian response, only the PMI and PMOI of the pregnant women in the normal ovarian response group were lower than those of the non-pregnant women. To compare the predictive value of the PFI and PMOI in IVF/ICSI outcomes, the patients were divided into four groups. The good-quality embryo rate and clinical pregnancy rate were highest in Group A (low PFI and low PMOI) and lowest in Group D (high PFI and high PMOI). In the two groups with discordant PFI and PMOI, namely Group B (low PFI and high PMOI) and Group C (high PFI and low PMOI), the good-quality embryo rate and clinical pregnancy rate were not significantly different.

**Conclusions:**

The PFI and PMOI had equal value in predicting clinical pregnancy outcomes in the normal ovarian response group undergoing the short-acting GnRH agonist long protocol. Each clinical centre can choose one of the indicators according to their actual situation in clinical practice and establish individual cut-off values for PFI and PMOI based on their own hormonal measurements.

## Background

During the process of controlled ovarian hyperstimulation (COH) using gonadotropins (Gn), serum progesterone (P) elevation is sometimes observed during the late follicular phase and on the day of human chorionic gonadotropin (HCG) administration [1]. The frequency of the serum P elevation varies according to the stimulation protocol. Studies have shown that the incidence of P elevation is 35% for the pituitary down-regulation protocol using the gonadotropin-releasing hormone (GnRH) agonist and 38% for the GnRH antagonist protocol [2]. There are many possible mechanisms of P elevation, including multiple follicles development, the high dose of exogenous Gn, the proliferation of granulosa cells and the increased activity of follicle-stimulating hormone (FSH)-stimulated granulosa cells and luteinizing hormone (LH)-stimulated theca cells [3]. Excessive P levels may affect the embryo quality and endometrial receptivity, reduce the embryo implantation rate, and thus reduce the clinical pregnancy rate [4]. Earlier studies did not show any association between P levels and pregnancy rates [5, 6], whereas more recent studies have reported a negative impact on pregnancy outcomes when serum P levels are increased [4, 7]. In this case, which indicator can we choose to predict pregnancy outcomes and subsequently decide to perform fresh embryo transfer (ET) or the “freeze-all” strategy?

There are many elevated P-related indicators for predicting pregnancy outcomes, including the serum P, P-to-oestradiol ratio (P/E_2_), P-to-follicle (≥ 14 mm in diameter on the HCG day) index (PFI), and P-to-mature oocyte index (PMOI). However, due to inconsistencies in study populations and COH protocols among the studies, these indicators are controversial. The serum P level on the day of HCG injection is the simplest indicator. Different studies have different definitions of thresholds for excessive P levels, ranging from 0.4 ng/ml to 3 ng/ml [8]. Moreover, the serum P levels during COH are associated with increased follicle count and oestradiol levels. Thus, researchers suggest that it would be better to define a ratio between the P level and ovarian response instead of using a single P level as a predictive indicator. Subsequent studies have compared the predictive values of P and P/E_2_, PFI, and PMOI, respectively [1, 9, 10]. However, none of these researches has included these four commonly used indicators in one study to compare their predictive efficacies. The objective of this study was to investigate which indicator was more accurate in predicting pregnancy outcomes of women undergoing the short-acting GnRH agonist long protocol by comparing the predictive value of the P-related indicators. Based on the above conclusions, clinicians can select the patients who would really benefit from delayed frozen embryo transfers.

## Methods

### Patients

In this retrospective study, we analysed the clinical data from 612 infertile women undergoing the short-acting GnRH agonist long protocol and fresh ET from January 2018 to December 2018 at the Reproductive and Genetic Medical Centre of Peking University First Hospital. Women with a history of endometrial lesions or endocrine diseases, including diabetes mellitus, thyroid disease, and hyperprolactinaemia, were excluded from this study. This study was approved by the Clinical Research Institutional Review Board of Peking University First Hospital.

### COH protocols

During the study period, all the participants were subjected to the short-acting GnRH agonist long protocol. The patients were treated with the short-acting GnRH agonist (0.1 mg per day) for 14–16 days from the mid-luteal phase of the last menstrual cycle for pituitary down-regulation. When the serum LH was lower than 5 IU/L, the serum E_2_ level was lower than 50 pg/ml and the endometrium thickness was less than 5 mm, which indicated down-regulation was achieved. Then, 150–300 IU of recombinant FSH was administered daily and the dose was adjusted according to the follicular development and hormone level of the patients. In addition, the GnRH agonist was reduced to 0.05 mg per day and continued until the day of HCG injection. Follicular development was regularly monitored by transvaginal ultrasound. Recombinant HCG was administered subcutaneously when the leading follicle was ≥ 18 mm in diameter. Serum samples were obtained on the morning of HCG injection for the serum P and E_2_ measurements. Transvaginal ultrasound was performed to determine the follicle count (≥ 14 mm in diameter). The oocytes were retrieved by transvaginal ultrasound-guided follicular aspiration within approximately 36 hours after HCG administration. The number of mature oocytes was observed in the embryo laboratory after oocyte retrieval. Luteal support was started on the day of oocyte retrieval (vaginal progesterone preparations, 90 mg per day). The oocytes were fertilized by conventional in vitro fertilization (IVF)/intracytoplasmic sperm injection (ICSI), and the embryos were transferred under abdominal ultrasound guidance on day 3 after oocyte retrieval. The transplantation was cancelled for the following reasons: (1) to prevent the occurrence of ovarian hyperstimulation syndrome (OHSS); (2) when no transplantable embryos were obtained; (3) to accumulate embryos; or (4) when the P levels were > 2.5 ng/ml on HCG day. HCG tests were performed on day 14 after ET, and if the result was positive, luteal support was continued. Transvaginal ultrasound was performed on day 28 after ET, and clinical pregnancy was defined as the presence of an intrauterine gestational sac or embryonic heartbeat.

The following criteria were used to define the ovarian response according to the oocyte yield [11]: poor ovarian response, oocyte yield < 4; normal ovarian response, oocyte yields ≥ 4 and ≤ 15; and high ovarian response, oocyte yields > 15. The P-related indicators were P (ng/ml), P/E_2_ [P (ng/ml) × 1000/E_2_ (pg/ml)], the PFI, and the PMOI.

### Statistical analysis

All the analyses were performed using the Software Package for Social Sciences (SPSS) version 13.0 for Windows. All the normally distributed measurement data are expressed as the means ± standard deviation (SD). Comparisons between two groups were analysed using independent sample t-tests; comparisons among multiple samples were analysed using variance analyses; and intergroup multiple comparisons were analysed using the Bonferroni correction. A *P* < 0.05 was considered statistically significant. A receiver operating characteristic (ROC) curve analysis was performed to compare the predictive values of the progesterone-related indicators for pregnancy outcomes. The highest value for the area under the curve (AUC) was determined.

## Results

A total of 612 patients were enrolled in the study, and 274 women (44.77%) had a clinical pregnancy. As shown in Table 1, there was no significant difference in age, infertility period or basal FSH between the pregnant and non-pregnant groups. The antral follicle count (AFC) of the pregnant group was significantly higher than that of the non-pregnant group.

**Table 1:** Comparison of clinical data of patients in different groups

Parameters	Pregnant group (*n* = 274)	Non-pregnant group (*n* = 338)	*P* value
Age (years)	32.22 ± 4.03	32.61 ± 4.66	0.266
Infertility period(years)	3.33 ± 2.53	3.22 ± 2.32	0.570
Basal FSH (mIU/mL)	7.34 ± 1.82	7.63 ± 2.21	0.085
No. of AFCs (n)	12.74 ± 5.73	11.48 ± 5.41	0.006*
P on HCG day (ng/ml)	1.06 ± 0.45	1.02 ± 0.47	0.683
P/E_2_	0.40 ± 0.26	0.43 ± 0.25	0.141
PFI	0.13 ± 0.08	0.15 ± 0.10	0.002*
PMOI	0.17 ± 0.12	0.21 ± 0.18	0.002*

*Indicates a significant difference (*P* < 0.05)

Among the 612 infertility patients included in the study, there was no significant difference in serum P level or P/E_2_ between the pregnant and non-pregnant groups. The PFI and PMOI of the pregnant group were significantly lower than those of the non-pregnant group (*P* = 0.002, 0.002, respectively). The results are shown in Table 1.

**Fig. 1:**
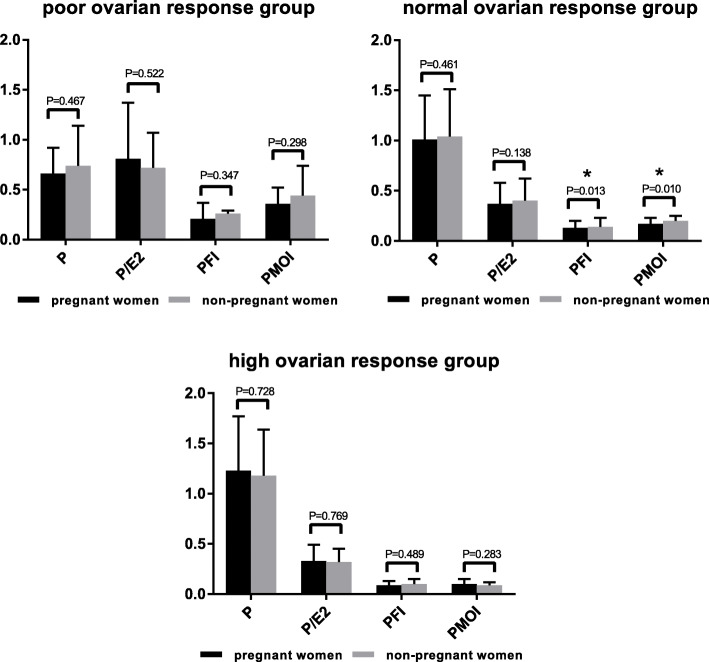
Progesterone-related indicators and pregnancy outcomes in women with different ovarian responses. The serum P levels increased with the number of oocytes retrieved, and they were lowest among women with a poor ovarian response and highest among women with a high ovarian response. In the normal ovarian response group, there was no significant difference in the serum P or P/E_2_ between the pregnant and non-pregnant groups. The PFI and PMOI of the pregnant group were significantly lower than those of the non-pregnant group

According to the stratified analysis of the ovarian response, among the poor ovarian response and high ovarian response groups, there was no significant difference in any P-related indicator between the pregnant and non-pregnant groups. In the normal ovarian response group, there was no significant difference in the serum P level or the P/E_2_ between the pregnant and non-pregnant groups. The PFI and PMOI of the pregnant group (0.13 ± 0.07, 0.17 ± 0.11) were significantly lower than those of the non-pregnant group (0.14 ± 0.09, 0.20 ± 0.15) (*P* = 0.013, 0.010, respectively). The results are shown in Fig. 1.

**Fig. 2:**
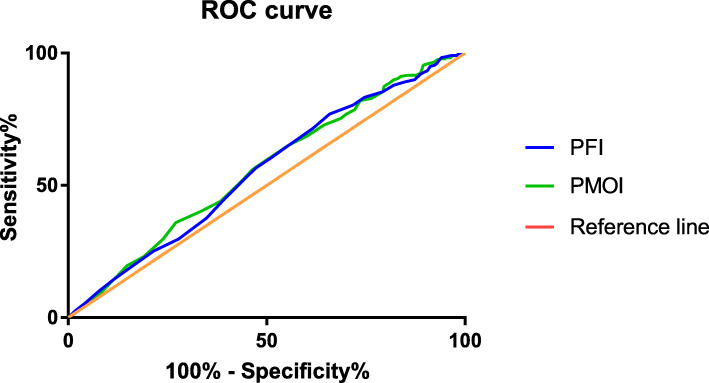
Predictive efficacies of progesterone-related indicators for pregnancy outcomes among women with normal ovarian responses

In the normal ovarian response group, according to the ROC analysis, the PFI and PMOI had some value in predicting clinical pregnancy outcomes, but the predictive efficacy was poor. The AUCs of the PFI and PMOI were 0.554 (95% confidence interval [CI]: 0.504–0.603) and 0.560 (95% CI: 0.511–0.609) (Fig. 2). The optimal cut-off value for the PFI and PMOI were 0.17 and 0.18.

To compare the predictive value of the PFI and PMOI in IVF/ICSI outcomes, we studied cases with PFI < 0.17 and PMOI < 0.18 (Group A), cases with PFI < 0.17 and PMOI ≥ 0.18 (Group B), cases with PFI ≥ 0.17 and PMOI < 0.18 (Group C) and cases with PFI ≥ 0.17 and PMOI ≥ 0.18 (Group D). As shown in Table 2, the good-quality embryo rate and clinical pregnancy rate were highest in Group A and lowest in Group D. In the two groups with discordant PFI and PMOI values, namely, Group B (low PFI and high PMOI) and Group C (high PFI and low PMOI), the good-quality embryo rate and clinical pregnancy rate were not significantly different.

**Table 2:** Pregnancy outcomes of patients according to the PFI and PMOI levels

Parameters	Group A PFI < 0.17 and PMOI < 0.18 (*n* = 273)	Group B PFI < 0.17 and PMOI ≥ 0.18 (*n* = 98)	Group C PFI ≥ 0.17 and PMOI < 0.18 (*n* = 42)	Group D PFI ≥ 0.17 and PMOI ≥ 0.18 (*n* = 110)	*p* value
Good-quality embryo rate (%)	43.55% (885/2032)	34.77%^▲^ (169/486)	36.48%^▲^ (116/318)	34.02% (180/529)	< 0.001
Clinical pregnancy rate (%)	52.38% (143/273)	40.82%^▲^(40/98)	50.00%^▲^ (21/42)	31.82% (35/110)	0.002

^▲^There were no statistically significant differences between Group B and Group C (*p* = 0.651;0.355)

## Discussion

During COH in assisted reproductive technology, the oestrogen levels are far above normal physiological levels due to the development of multiple follicles. Some patients may also have elevated serum P levels during the late follicular phase. Excessive P can affect the embryo implantation rate and clinical pregnancy rate [12, 13]. This result primarily occurs because excessive P causes asynchronization between the endometrium and embryo development [14]. Therefore, if the P level is too high during the late follicular phase, then the cancellation of the fresh ET cycle and the freezing all the embryos are recommended [15]. Compared with that of the fresh ET cycle, the pregnancy rate of the frozen ET (FET) cycle is higher [16]. Some studies have shown that excessive P can also affect the embryo quality [17, 18]. Vanni *et al*. studied 986 GnRH antagonist IVF/ICSI cycles, and the results demonstrated that a high-quality blastocyst rate was negatively correlated with high P levels [17]. In cases with high P levels, the “freeze-all” strategy is not the best way to solve the problem because the embryo utilization rate is significantly reduced [18]. A recent study showed that excessive P is neither associated with impaired embryonic development nor increased rates of aneuploidy. Patients can expect to optimize their implantation potential by employing an FET cycle strategy [19]. Therefore, it is controversial as to whether excessive P impairs the quality of the embryo, but it is certain that excessive P causes embryo-endometrium asynchrony, thus affecting the pregnancy outcome of the fresh ET cycle. In this case, we want to determine if the specific indicator choose can help to predict pregnancy outcomes and subsequently decide to perform fresh ET or the “freeze-all” strategy.

There are many P elevation-related indicators for predicting pregnancy outcomes. The simplest indicator is the serum P level on the day of HCG injection. Li et al. proposed that serum P levels on the day of HCG injection can predict IVF pregnancy outcomes, but the predictive efficacy is poor (AUC = 0.599). For patients with P levels exceeding 6.0 nmol/L, the cancellation of fresh ET and the freezing all the embryos are recommended [20]. It is well known that the serum P level during COH is associated with the ovarian response and increases as the number of follicles and oestradiol level [21]. Therefore, the P/E_2_ may be more effective at predicting the pregnancy outcome than the serum P alone [9], but its predictive efficacy is still poor [22, 23]. Shufaro et al. studied women with a normal ovarian response who were undergoing GnRH agonist and GnRH antagonist protocols, and the results demonstrated that the PFI can better predict the clinical pregnancy rate than the serum P [24]. Roque et al. performed a stratified analysis of patient ages during treatment with the GnRH antagonist protocol and found that a PFI with a cut-off value of 0.075 is a good predictor for IVF outcome among patients of all ages [1]. Simon et al. found that the PMOI was negatively correlated with the live birth rate and the implantation rate. The PMOI seems to be more predictive than the serum P level in terms of IVF outcomes [10]. The researchers also believe that the PFI is affected by the ultrasound equipment and the experience of the examiner and that the PMOI is more reproducible. In addition, studies have indicated that for patients with a PMOI exceeding 0.32, a fresh cycle transfer is recommended [25].

Although there are many studies on the predictive value of P-related indicators for IVF/ICSI pregnancy outcomes, none of these researches has included these four commonly used indicators in one study to compare their predictive efficacies. The results of this study showed that there was no significant difference in serum P or P/E_2_ between the pregnant group and the non-pregnant group for cases in which the P was < 2.5 ng/ml. The PFI and PMOI of the pregnant group were significantly lower than those of the non-pregnant group. Our findings were in accordance with previous studies by Shufaro et al. and Simon et al., who found that the PFI and PMOI were better correlated with the IVF outcome than the serum P levels during fresh ET [10, 24]. According to the stratified analysis of the ovarian response, the results showed that the PFI and PMOI of the pregnant group were lower than those of the non-pregnant group only in the normal ovarian response group, and the differences were statistically significant. This finding may have occurred because factors affecting pregnancy outcomes are more complicated among patients with poor ovarian response or high ovarian response, including the number of retrieved oocytes and higher levels of oestrogen such that the effect of elevated P levels on pregnancy outcomes is relatively small. The results showed that in cases when progesterone < 2.5 ng/ml, the PFI and PMOI had some value in predicting clinical pregnancy outcomes in the normal ovarian response group. The optimal cut-off values for the PFI and PMOI were 0.17 and 0.18 in predicting the clinical pregnancy outcomes, which were inconsistent with the previous results. Roque et al. reported that the cut-off value of PFI was 0.075 with the GnRH antagonist protocol [1]. Simon et al. found that the cut-off value of PMOI was 0.167 with the GnRH antagonist protocol [10], and Aflatoonian et al.. found that the cut-off value of PMOI was 0.32 with the GnRH agonist protocol, GnRH antagonist protocol and microdose protocol [25]. We speculate that differences in the cut-off values may be due to inconsistencies in COH protocols among studies. Additionally, studies attempting to establish cut-off values using hormonal measurements may be influenced by the different measurement techniques in use or by the labs in which they are measured [1]. These variations may be responsible for the differences between the cut-off values reported by the different studies. Thus, we recommend that each clinical centre establish individual cut-off values for the PFI and PMOI based on their own hormonal measurements. When comparing the PFI and PMOI, the prediction efficiency was approximately equal. Therefore, during clinical practice, each clinical centre can choose one of the indicators according to their actual situation. For example, some centres do not always know the number of follicles (≥ 14 mm) on HCG day; in these cases, the PMOI can be used.

The limitation of this study is that the cut-off value of serum P used in our study was based on prior publications [20], and our reproductive centre recommends cancelling fresh ET when the P exceeds 2.5 ng/ml. Therefore, this study was conducted primarily to compare the predictive value of P-related indicators for pregnancy outcomes of women undergoing the short-acting GnRH agonist long protocol when their *P* level is < 2.5 ng/ml. To date, there is still no consensus on the exact cut-off value of serum P to determine the fresh ET or “freeze-all” strategy.

Another limitation is that there are fewer patients in Group B and Group C when grouping statistics is performed. In fact, the incidence of discordance between PFI and PMOI is quite low in clinical practice. The result can be further verified by expanding the sample size in the future.

## Conclusions

In conclusion, in cases with *P* < 2.5 ng/ml, the PFI and PMOI had some value in predicting clinical pregnancy outcomes in the normal ovarian response group undergoing the short-acting GnRH agonist long protocol. In comparing the PFI with the PMOI, the prediction efficiency was approximately equal. Each clinical centre can choose one of the indicators according to their actual situation in clinical practice and establish individual cut-off values for PFI and PMOI based on their own hormonal measurements. If the PFI or PMOI is greater than the cut-off value, the “freeze-all” strategy rather than fresh ET is recommended. Further randomized trials are required to validate the results found in this study.

## Data Availability

The datasets used and/or analysed during the current study are available from the corresponding author upon reasonable request.

## Abbreviations

AFC: Antral follicle count
AUC: Area under the curve
COH: Controlled ovarian hyperstimulation
ET: Embryo transfer
FSH: Follicle-stimulating hormone
Gn: Gonadotropins
GnRH: Gonadotropin-releasing hormone
HCG: Human chorionic gonadotropin
LH: Luteinizing hormone
OHSS: Ovarian hyperstimulation syndrome
P: Progesterone
P/E_2_: P-to-oestradiol ratio
PFI: P-to-follicle index
PMOI: P-to-mature oocyte index
ROC: Receiver operating characteristic
SD: Standard deviation
SPSS: Software Package for Social Sciences
